# Phylogenomics Reveals Clear Cases of Misclassification and Genus-Wide Phylogenetic Markers for *Acinetobacter*

**DOI:** 10.1093/gbe/evz178

**Published:** 2019-08-12

**Authors:** Valeria Mateo-Estrada, Lucía Graña-Miraglia, Gamaliel López-Leal, Santiago Castillo-Ramírez

**Affiliations:** Programa de Genómica Evolutiva, Centro de Ciencias Genómicas, Universidad Nacional Autónoma de México, Cuernavaca, México

**Keywords:** phylogenomics, *Acinetobacter*, bacterial species, evolutionary biology

## Abstract

The Gram-negative *Acinetobacter* genus has several species of clear medical relevance. Many fully sequenced genomes belonging to the genus have been published in recent years; however, there has not been a recent attempt to infer the evolutionary history of *Acinetobacter* with that vast amount of information. Here, through a phylogenomic approach, we established the most up-to-date view of the evolutionary relationships within this genus and highlighted several cases of poor classification, especially for the very closely related species within the *Acinetobacter calcoaceticus–Acinetobacter baumannii* complex (Acb complex). Furthermore, we determined appropriate phylogenetic markers for this genus and showed that concatenation of the top 13 gives a very decent reflection of the evolutionary relationships for the genus *Acinetobacter.* The intersection between our top markers and previously defined universal markers is very small. In general, our study shows that, although there seems to be hardly any universal markers, bespoke phylogenomic approaches can be used to infer the phylogeny of different bacterial genera. We expect that ad hoc phylogenomic approaches will be the standard in the years to come and will provide enough information to resolve intricate evolutionary relationships like those observed in the Acb complex.

## Introduction

The biological species concept creates challenges to many organisms, from large mammals to bacteria (Hey 2001); but bacteria are particularly affected because the phenotypic characters that can be used for their classification are limited. Nonetheless, bacterial species designation has a vital role in clinical environments, food industry, agriculture, bioremediation, public health, environmental sciences, and biosafety (Gevers et al. 2005). From a clinical point of view, the description and classification of bacteria are of great importance when it comes to identifying pathogenic agents, which determines epidemiological characteristics useful to its treatment and prevention (Godreuil et al. 2005). Also, cataloging species can reveal clues about the evolutionary forces behind the emergence, transformation, and extinction of bacterial lineages; and even the role of different mechanisms of genetic differentiation and the course of adaptation to new niches (Fraser et al. 2009).

The genus *Acinetobacter*, which belongs to the order *Pseudomonadales* within the γ-Proteobacteria, is a genus of Gram-negative, oxidase-negative, and strictly aerobic bacteria. The genus includes pathogenic and nonpathogenic species (de Berardinis et al. 2009). *Acinetobacter* spp. have been increasingly recognized as important nosocomial pathogens involved in hospital outbreaks; particularly in intensive care units, where they quickly develop resistance even to the most potent antimicrobials (Turton et al. 2010; Antunes et al. 2014). Furthermore, they are very abundant in natural environments, including soils, water, oceans, sediments, polar regions, and contaminated sites (Al Atrouni et al. 2016). Additionally, these species have physiological characteristics associated with important microbiological aspects such as biofilm formation, quorum sensing, oxidative stress, and resistance to antibiotics (Jung and Park 2015). At the time of writing, 60 species with valid names could be found in the List of Prokaryotic Names with Standing in Nomenclature (LPSN, http://www.bacterio.net/; last accessed August 22, 2019), and more are waiting for validation (https://apps.szu.cz/anemec/Classification.pdf; last accessed August 22, 2019). Although the genus description dates back to 1954 (Brisou and Prevot 1954), most species have been described in the last 10 years, at which time many genomospecies have been resolved and named; this goes to show the rapid development of the *Acinetobacter* taxonomy, which in turn reflects the methodological improvements in bacterial systematics over the last years (Caputo et al. 2019). Several species circumscription methods have been used for the *Acinetobacter* genus both phenotypic and genotypic. The problem of bacterial species identification affects this versatile genus to a large extent, as it includes a large number of named species but no simple technique for their proper identification (Dijkshoorn and Nemec 2008). At the phenotypic level, MALDI-TOF MS (matrix-assisted laser desorption/ionization time-of-flight mass spectrometry) is currently becoming the method of choice for the rapid identification of bacterial species in routine hospital diagnoses. However, this method cannot reliably differentiate between some closely related species, including those from the *Acinetobacter calcoaceticus–Acinetobacter baumannii* complex (Acb complex) (Šedo et al. 2013). Regarding the genotypic methods, the most prominent are DNA–DNA hybridization methods (DDH), different phylogenetic markers such as 16S rRNA, *rpoB* (La Scola et al. 2006), *recA* (Krawczyk et al. 2002), *gyrB* (Yamamoto et al. 1999; Teixeira et al. 2017) and, lately, whole genome sequencing (WGS) along with average nucleotide identity (ANI) methodology have been applied (Nemec et al. 2017; Hu et al. 2018). Of note, from a clinical point of view, the proper identification of a species is of paramount importance, given that very closely related species can have very different antibiotic resistance phenotypes. Thus, to ensure an adequate antimicrobial treatment a reliable species assignation can be very useful.

Numerous completed genome sequences are now available for *Acinetobacter* spp. opening up the possibility of using whole genome approaches to infer species relationships. Moreover, next-generation sequencing technologies have enabled sequencing genomes of multiple strains within a species or even a population, making it possible to untangle the level of intraspecies variation in the genus (Graña-Miraglia et al. 2017). Despite the great number of genomes available for this genus, no recent study has tried to infer the evolutionary relationships for the species within the *Acinetobacter* genus.

Here, we use a phylogenomic approach to provide the latest view of the phylogenetic relationships for the *Acinetobacter* spp., highlighting several cases of misclassification, and to produce a list of well-suited genes for species assignation in the genus. This set of genes was chosen for meeting the most valuable requirements in a phylogenetic marker; high genetic diversity, universality across the genus, no signs of recombination, and a genomic stable context.

## Materials and Methods

### Database

We built one of the most comprehensive genome databases for the genus *Acinetobacter* to date. A total of 230 genomes of almost all of the *Acinetobacter* species described were downloaded from NCBI in November 2018. For each species, we included a maximum of ten either draft or complete genomes and, when, possible at least one type strain. Also, variation within species was taken into account including different sequence types (ST) when possible. We analyzed the completeness and contamination of each genome with CHECKM (Parks et al. 2015), and the genomes that did not meet the requirements of less than 5% contamination and more than 95% completeness were excluded from the database. Species with few genomes at NCBI, *Acinetobacterpuyangensis, Acinetobacterqinfengensis, Acinetobacterpragensis*, *Acinetobacterbohemicus, Acinetobacterkyonggiensis*, and *Acinetobactermarinus* did not meet the quality requirements and were left out of the study. These analyses led to a database of 214 genomes representing 51 different *Acinetobacter* species (see supplementary table 1, Supplementary Material online), which were (re) annotated using PROKKA version 1.12 (Seemann 2014).

### Homologous Groups and Phylogenetic Reconstruction

To construct orthologous groups, we run BlastP of *A. baumannii* ACICU proteome against the whole database as described in Graña-Miraglia et al. (2018). We selected hits with ≥40% identity, ≥60% of alignment coverage, and with an e-value cutoff of 1.0e-30, as we did in a previous study analyzing the genus *Staphylococcus* (Graña-Miraglia et al. 2018). We created homologous groups (HG) that had only one gene per genome, which we will refer to as single gene families (SGF) from now on; we found 305 SGFs. Each SGF was aligned with Fast Statistical Alignment version 1.15.9 employing the option –nucprot to align in frame (Bradley et al. 2009). We further tested every SGF for recombination using PhiTest implemented via PhiPack (Bruen et al. 2006), setting the window parameter to 50 nucleotides, and 50 SGFs showed recombination signals. We concatenated all the nonrecombinant SGF alignments and built a tree with RAxML version 8.2.4 with 20 independent inferences from 20 different maximum parsimony trees using GTRGAMMAIX model (Stamatakis 2014), this was our Species Tree. Similarly, we constructed trees as described above but with ten different randomized maximum parsimony trees for each SGF, for the top-ranked genes concatenated alignments and for the concatenated randomly chosen genes. The Shimodaira–Hasegawa (SH) topology test and Robinson–Fould distance (RFD) against the Species Tree were implemented via RAxML version 8.2.4 with the options –f H and –f r, respectively.

### Ranking of Phylogenetic Markers, ANI Analysis, and Screening in Environmental Samples

The SGF were ranked according to the percentage of shared bipartitions (ShBip) with the Species Tree and to their nucleotide diversity (π) values, which were obtained with “pegas” in R (Paradis 2010). SGF were ranked in decreasing order according to ShBip and then according to π. Functional annotation for each gene was corroborated in UniProt. We also performed an ANI analysis to estimate the relatedness of the genomes. For this purpose, we ran pyani with MUMmer (ANIm) (Pritchard et al. 2016). The genome pairs with more than 95% of identity were considered to belong to the same species (Goris et al. 2007). The results were visualized with Pheatmap R library. To evaluate the utility of our top marker for species assignation in metagenomes, we downloaded 32 samples from a large freshwater metagenome (The Anacostia river data set, PRJNA498951), reported to have the presence of *Acinetobacter* species. We used TrimGalore (to trim the reads) and fastqc (to check the quality) to process the data. This shotgun metagenomic data set has 571,885,812 pair-end reads on which we screened for the presence of two *Acinetobacter* spp. using our top markers and employing the SRST2 tool (Inouye et al. 2014).

### Gene Composition Analysis

We used ROARY (Page et al. 2015) to build a matrix of gene content for all species in the genus. We modified default identity (45%) and coverage (60%) parameters to fit a genus analysis. We built a Euclidean distance matrix using the dist function in R and from this, a Neighbor-Joining (NJ) tree using “ape” package (Paradis et al. 2004). We also built a Bray–Curtis dissimilarity index matrix that was analyzed using principal coordinates analysis (PCoA) with “vegan” in R as in Tu and Lin (2016). A correlation matrix was created employing the cor() function in R based on an initial gene content matrix; where the gene content matrix was constructed as in our previous study (Graña-Miraglia et al. 2017).

## Results

### Establishing the True Evolutionary Relationships for *Acinetobacter* spp.

**Fig. 1.**
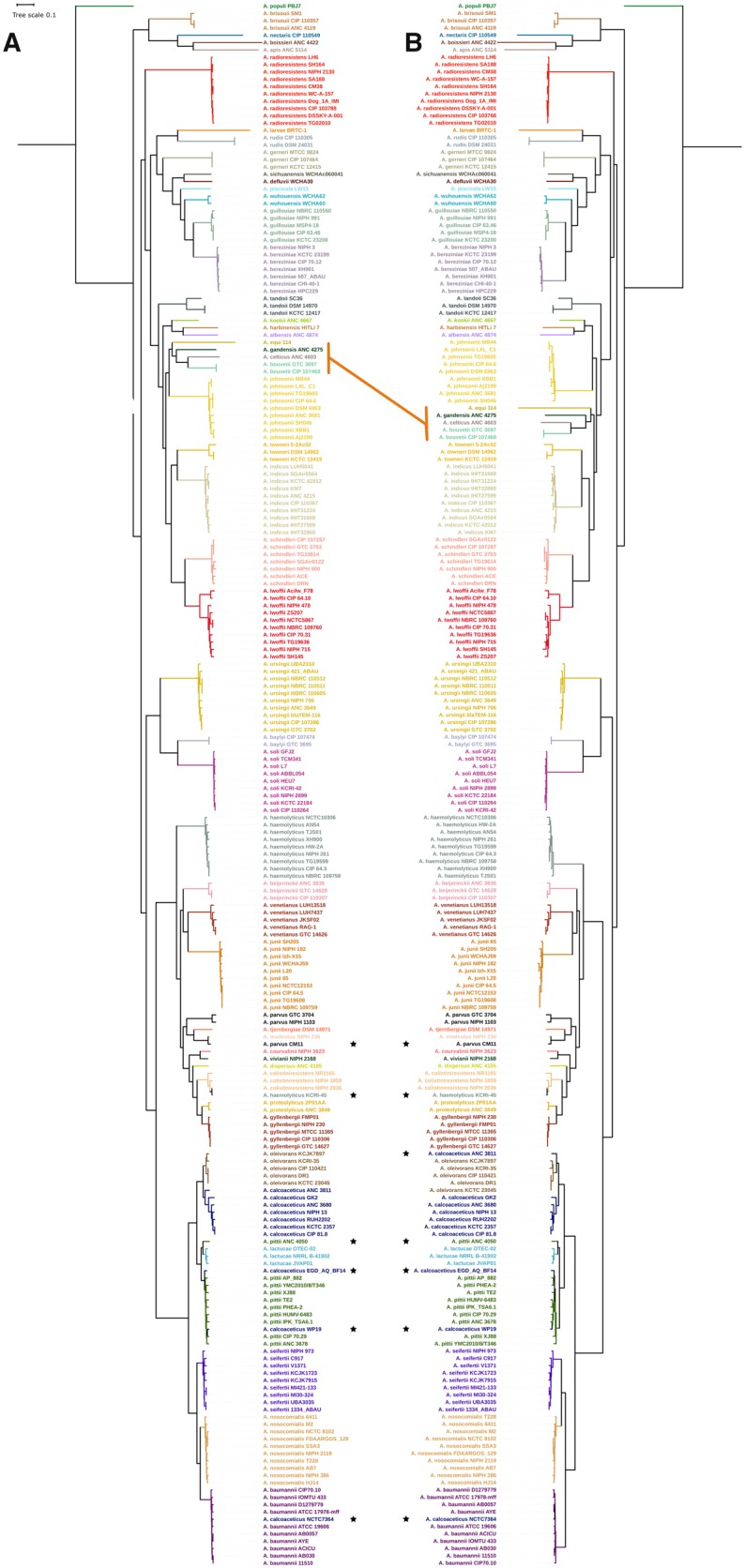
—(*A*) Maximum likelihood phylogenetic reconstruction of the species tree for the genus *Acinetobacter* based on the concatenated alignment of 255 nonrecombinant SGF. (*B*) Maximum likelihood phylogenetic reconstruction based on the 13 top-ranked genes for species assignation (table 1). (*A* and *B*) Strains belonging to the same species are colored equally, asterisks show misclassified strains. The orange line highlights one major topological difference between the two trees; this difference involves the clade composed by *Acinetobacter equis*, *Acinetobacter gandensis*, *Acinetobacter celticus*, and *Acinetobacter bouvetii*. Tree scale shows substitutions per site.

The genus *Acinetobacter* comprises 60 species with validly published names and at least five more could be added soon (http://apps.szu.cz/anemec/Classification.pdf). As of November 2018, at least 55 species had one or more genomes publicly available. The number of *Acinetobacter* species described has grown exponentially in recent years but the number of publicly available genomes for each species is still very uneven. Therefore, in creating our *Acinetobacter* genome database, for species with a large number of sequenced strains we only included ten genomes and when the number of genomes for a species was lower than ten, we included as many as available. Type strains were included, when possible, and also different genotypes as per Multi Locus Sequence Typing scheme for *A. baumannii* and *Acinetobacterhaemolyticus*. Only high-quality genomes were included (see Materials and Methods) and in total 214 complete genome sequences, comprising 51 species (see supplementary table 1, Supplementary Material online), were considered for the analyses. To reconstruct the phylogenetic relationships of the *Acinetobacter* species, we used SGF without recombination signals (see Materials and Methods) (Castillo-Ramírez and González 2008) as a proxy of orthologous genes. We found 255 nonrecombinant SGFs and built a phylogeny on the concatenated alignment of these 255 SGFs and rooted the phylogeny using *Moraxella atlantae* and *Moraxella catarrhalis*, two species of a closely related genus. Figure 1*A* shows this phylogeny, which had very good support for most of the clades, as most of the bootstrap values were higher than 80%.

Most of the strains previously assigned to a given species were grouped into monophyletic clades yet some clear exceptions were poorly classified (see asterisks, fig. 1*A*). Particular cases are highlighted below, but it is worth mentioning that most of them correspond to strains whose genomic features like G + C content or genome size did not match those of the assigned species. We obtained similar clades to those found in a previous study using just a few genomes (Touchon et al. 2014); however, the clades in our study also include other species whose kinship with the rest of the genus was not shown previously. The species branching deeper in the genus is *Acinetobacterpopuli* and there is also a basal clade formed by *Acinetobacterapis, Acinetobacterboissieri, Acinetobacternectaris*, and *Acinetobacterbrisouii*. Furthermore, *Acinetobacterradioresistens* is also placed in a basal position. The Acb complex is a taxonomic group defined by the inability to properly distinguish phenotypically the species that conform it (Gerner-Smidt et al. 1991) and these species are *A. baumannii, Acinetobacterpittii, Acinetobacternosocomialis, A. calcoaceticus*, and *Acinetobacterseifertii*. The group has been previously described as a monophyletic clade (Touchon et al. 2014) and our phylogeny shows that *Acinetobacteroleivorans* and *Acinetobacterlactucae*, quite recently described (Kang et al. 2011; Rooney et al. 2016), cluster within the Acb complex even though were supposed to cluster outside the Acb complex. We found various misclassified strains within the Acb complex (see asterisks in fig. 1*A*). For instance, two *A. calcoaceticus* strains (WP19 and EGD_AQ_BF 14) were classified as *A. pittii* according to our phylogeny; additionally, *A. calcoaceticus* NCTC 7364 clustered within *A. baumannii.* This is not surprising given that classification issues are common in the Acb complex. Also the *A.pittii-*like strain ANC 4050 was grouped with the closely related species *A. lactucae*, but judging by the long-branch length, *A. pittii-*like strain ANC 4050 could be classified as a new, different species within the Acb complex. Another case of misclassification is *A. haemolyticus* strain KCRI-45, which according to our phylogeny the strain actually belongs to *Acinetobactercolistiniresistens* species. Furthermore, a strain previously classified as *Acinetobacterparvus* CM11 was shown to be *Acinetobactermodestus* species. This strain (*A. parvus* CM11) was classified using 16S rRNA, *rpoB*, and *gyrB* (Saffarian et al. 2015), but identity percentages estimated from *gyrB* and *rpoB* alignments of *A. parvus* CM11 and the *A. parvus* type strain indicate that the two strains cannot belong to the same species (85.47% and 95%, respectively). The previous cases demonstrate that several cases of poor classification have occurred within this genus.

The species assignation recovered from the phylogeny was confirmed with an ANI analysis (see supplementary fig. 1, Supplementary Material online). The strains grouped as monophyletic clades in the Species Tree show ANI values above 95%, which is the threshold for species designation (Richter and Rosselló-Móra 2009). Moreover, misclassification cases are also corroborated by ANI values; for instance, the *A.pittii-*like strain, ANC 4050, does not group with any other species and the highest ANI value observed for this strain is 94% with an *A. lactucae* strain, supporting the idea that this could be a different species also part of the Acb complex. In summary, we obtained a robust phylogeny for this genus and found some misclassified strains, which show that misclassification usually occurs when nonreliable methods for species assignation are used.

### Gene Content Dissimilarity Is Not Useful for Species Delineation in *Acinetobacter*

**Fig. 2.**
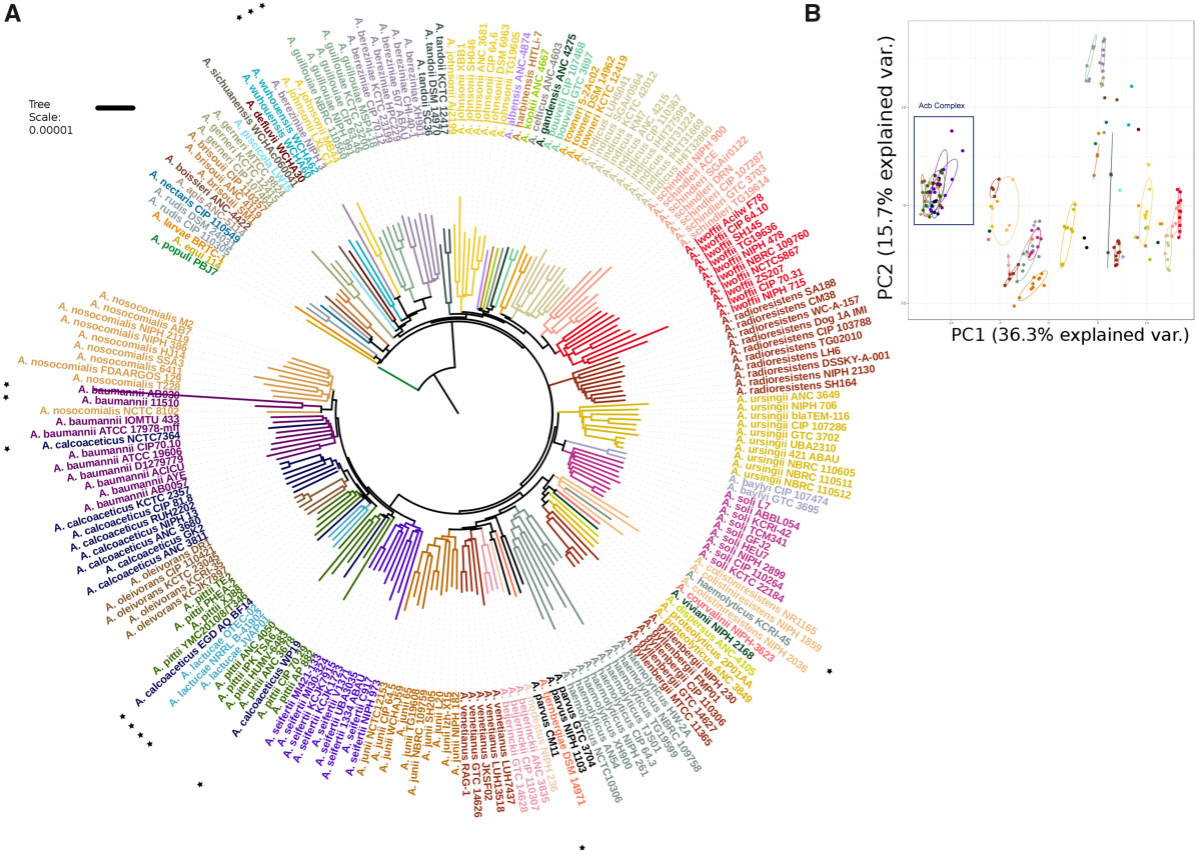
—(*A*) NJ approach analysis of gene composition. Strains belonging to the same species are colored equally, asterisks show misclassified strains and strains that are grouped differently in the Species Tree phylogeny. (*B*) PCoA approach. Biplot Dots share color code with (*A*) Acb complex is denoted with a black rectangle.

It has been proposed that shared gene content between genomes is quantitatively determined by phylogeny (Snel et al. 1999) and that genomic fluidity is linked with microbial taxonomy; therefore, gene content dissimilarity can distinguish between closely related bacterial species (Tu and Lin 2016). Also, the identification of unique genes specific to each taxonomic rank has been used for assigning the bacterial taxonomy (Gupta and Sharma 2015). We calculated the gene repertoire for the *Acinetobacter* species and obtained a total of 39,595 HG (26,221 excluding unique genes) and built a gene composition profile for every genome. These data were analyzed with two different methodologies; on the one side, a Euclidean distance matrix was estimated from these data and the relationships were determined through an NJ tree (fig. 2*A*). On the other side, the Bray–Curtis dissimilarity index was estimated (excluding unique genes) and grouping was established through a PCoA as in Tu and Lin (2016) (fig. 2*B*).

Clearly, the grouping obtained with the NJ approach reflects kinship between strains, as it recovered the monophyletic groups for most of the species, although the topology depicting the evolutionary relationships between species appears to be significantly different to the Species Tree topology as per the SH test (*P* value <0.05). Some of the poorly classified strains detected with the Species Tree and ANI analysis could also be reassigned with the NJ topology but notably the relationships in the Acb complex are very dissimilar. The PCoA analysis also revealed issues with the Acb complex species, as the dots representing Acb complex strains are completely overlapped. Furthermore, the gene content dissimilarity values of these strains were lower than 0.2, which is the suggested cutoff value for species assignation by Tu and Lin (2016). Furthermore, two strains (AB030 and 11510) belonging to *A. baumannii* (fig. 1*A*) were placed within the *A. nosocomialis* cluster in the NJ approach. Notably, we have previously observed that the AB030 strain has a gene composition radically different from the rest of *A. baumannii* genomes (Graña-Miraglia et al. 2017). Moreover, *A. pittii* strains did not form a monophyletic group and were located in three different points in the tree (see fig. 2*A* dark green branches). To better understand the gene content dissimilarity across the genus, we created a gene content matrix considering all the genomes; this matrix was visualized using a heat map (see supplementary fig. 3, Supplementary Material online). From this analysis is clear that many species had considerable variation in gene content; one exception is *A.radioresistens* (small rectangle, supplementary fig. 3, Supplementary Material online), which actually show a very similar gene content. On the other hand, from this analysis is clear that species from the Acb complex (big rectangle, supplementary fig. 3, Supplementary Material online) have a rather similar gene content, which might help to explain why this group is not well differentiated in PCoA analysis. To sum up, the use of gene content variation to conduct taxonomic assignation is not reliable for this genus; this is especially true for the Acb complex, where species are not well defined by gene composition.

### Adequate Phylogenetic Markers for the Genus

**Fig. 3.**
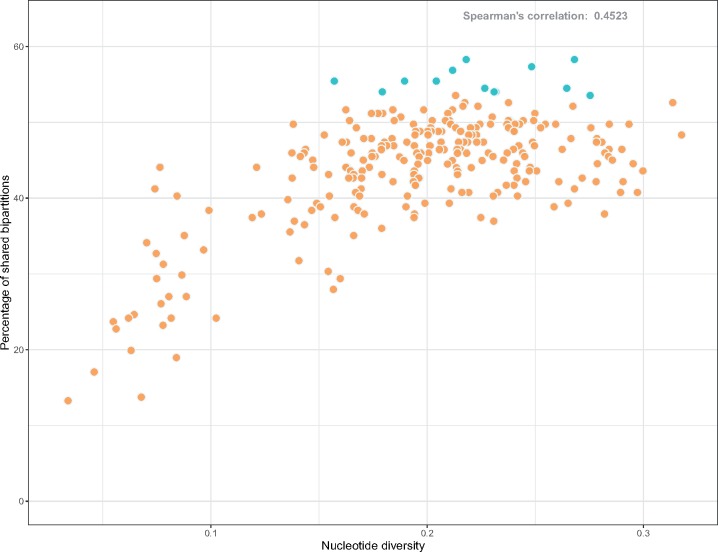
—The nucleotide diversity of every SGF plotted against the corresponding percentage of ShBip of the gene tree topology with the Species Tree. In turquoise, the top 13 genes in the ranked list of SGF are highlighted. Spearman correlation was estimated in 0.45.

To have an idea of the individual gene histories of the SGFs and see how they compare with the Species Tree, we built individual gene trees for all the 255 SGF used in the Species Tree and compared their topologies with the Species Tree topology through the SH test and RFD. None of the SGF topologies differ significantly from the species tree according to the SH test (*P* < 0.05), but RFDs were found to vary widely, between 176 and 366. The RFD is the number of bipartitions that are different between the two topologies being compared and it depends on the tree size (number of bipartitions). Here, to standardize RFD we use the percentage of shared bipartitions as a measure of similarity between two topologies. The set of SGFs showed on average a 43.91% of shared bipartitions with the species tree, being the lowest 13.27% and the highest 58.29%. There was no gene tree topology identical to that of the Species Tree. We also estimated the nucleotide diversity (π) of the nonrecombinant SGFs and found that the mean π of all the SGFs was 0.19 ± 0.05; furthermore, tree topologies of genes with high levels of π show higher similarity with the Species Tree (Spearman correlation = 0.4523) (fig. 3). This has been previously observed in the genus *Staphylococcus* (Graña-Miraglia et al. 2018) and it is due to the resolution improvement that comes with increasing levels of genetic diversity; along those lines, the smallest levels of topological congruence with the Species Tree were produced by SGFs with π values below 0.19. The nucleotide diversity (π) of the SGF can and has been used as a measure of the phylogenetic power of the genes (Cooper and Feil 2006).

**Fig. 4.**
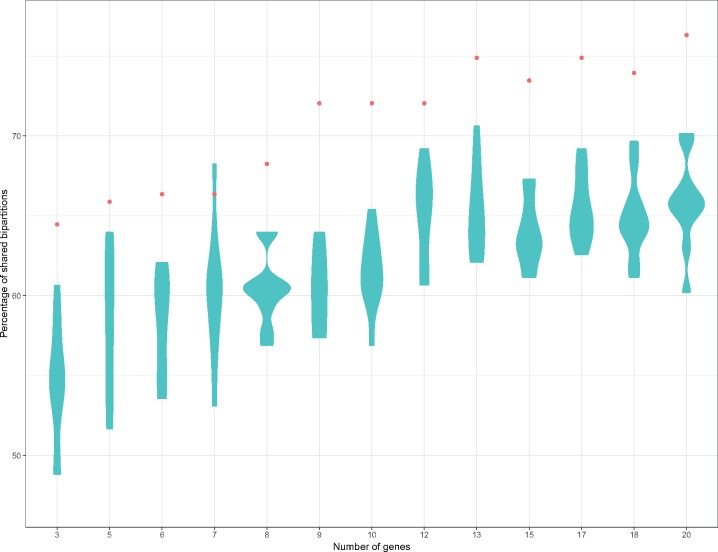
—Percentage of shared bipartitions with the Species Tree for the trees estimated for the concatenated alignments of the top 3, 5, 6, 7, 8, 9, 10, 12, 13, 15, 17, 18, 20 genes (red dots) and for 3, 5, 6, 7, 8, 9, 10, 12, 13 15, 17, 18, 20 randomly chosen genes.

It is well established that a tree topology built from a single locus is not likely to agree with that of the species tree (reflecting the evolutionary history of the species), but this probability increases when several *loci* are used (Pamilo and Nei 1988). For instance, a common practice when assigning strains to a species is to sequence a couple of phylogenetic markers, being a very frequent combination the 16S rRNA and a protein-coding gene such as *rpoB* or *gyrB* (Choi et al. 2013; Wang et al. 2015). Therefore, we ranked our 255 SGF according to the topology similarity with the Species Tree to have an idea of a decent set of phylogenetic markers for the genus *Acinetobacter*; π was also taken into account and genes with π above the SGF mean were preferred over those with π below the mean. Then, we concatenated the alignments of the top 3, 5, 6, 7, 8, 9, 10, 12, 13, 15, 17, 18, 20 SGFs, built phylogenies and compare them to the Species Tree (fig. 4). As expected, we observed an increase in topology similarity when increasing the number of loci used for the phylogenetic estimation. Furthermore, the concatenated alignments of randomly chosen (from the 255 SGFs) 3, 5, 6, 7, 8, 9, 10, 12, 13, 15, 17, 18, 20 genes were tested and even though the percentage of shared bipartitions increases with loci number, in all cases but one it never reaches the similarity obtained by the top-ranked genes (fig. 4). We noted that with just the 13 top SGFs a very good reflection of the evolutionary relationships was reached (75% of shared bipartitions); we acknowledge that settling on the minimum number of genes reaching 75% of shared bipartitions was a judgment call and, as such, is not meant to be definitive or exhaustive. The top 20 genes are described in table 1 and are not the most well-known phylogenetic markers for this genus. The top 13 genes in this ranking (table 1) had a percentage of shared bipartitions above 53, the mean π of the top 13 genes is 0.22, which is higher than the π estimated for the concatenated alignment of 255 SGF (mean = 0.201). The topology of the phylogenetic reconstruction based on their concatenated alignment retrieves most of the clades observed in the Species Tree (fig. 1*B*). Of note, there are three genes (*rpoB, recA*, and, *gyrB*) that have been extensively used for species delimitation in the genus *Acinetobacter*; however, out of the three only *recA* is part of the 255 SGFs but had low π value (0.179). *rpoB* is not within the 255 SGF as it did not fulfill the alignment length requirement in one strain (*Acinetobactersoli* L7 < 60% of the gene aligned), whereas *gyrB* could not be considered as single copy gene (40% similarity between *gyrB* and *parE* in *Acinetobacterlarvae*). Furthermore, both *rpoB* and *gyrB* had recombination signals. We did not analyze 16S rRNA performance in this study given as it has been previously shown for *Acinetobacter* that species delimitation with the current cutoff identity value (99%) is not possible (Chan et al. 2012; Wang et al. 2014).

**Table 1:** Top 20 Best Ranked Genes for Species Assignation in the Genus *Acinetobacter*

	Gene Name	RFD	%ShBip	π	UniProt Annotation
1	*NA*	176	58.2938	0.2681134	Site-specific recombinase
2	*miaA*	176	58.2938	0.2180549	tRNA dimethylallyltransferase
3	*pbp*	180	57.3460	0.2483083	Penicillin-binding protein 1B
4	*bamA*	182	56.8720	0.2117751	Outer membrane protein assembly factor BamA
5	*cbl*	188	55.4502	0.2042251	Cys regulon transcriptional regulator Cbl
6	*tqsA/AI-2*	192	54.5024	0.2645531	AI-2 transport protein TqsA
7	*hemA*	192	54.5024	0.2266403	Glutamyl-tRNA reductase
8	*YidC*	188	55.4502	0.1895524	Membrane protein insertase YidC
9	*ppsA*	188	55.4502	0.1570536	Phosphoenolpyruvate synthase/pyruvate phosphate dikinase
10	*mfd*	194	54.0284	0.2317331	Transcription-repair-coupling factor
11	*dxs* ^a^	194	54.0284	0.2309427	1-deoxy-d-xylulose-5-phosphate synthase
12	*NA*	194	54.0284	0.1792528	S1 RNA binding domain protein
13	*rlmD*	196	53.5545	0.2753780	23S rRNA (uracil(1939)-C(5))-methyltransferase RlmD
14	*gpsA*	196	53.5545	0.2131462	Glycerol-3-phosphate dehydrogenase [NAD(P)+]
15	*lpxK*	200	52.6066	0.3135703	Tetraacyldisaccharide 4′-kinase
16	*NA*	200	52.6066	0.2376158	Class II glutamine amidotransferase
17	*rluD* ^b^	200	52.6066	0.2173651	Pseudouridine synthase
18	*cdsA*	202	52.1327	0.2674116	Phosphatidate cytidylyltransferase
19	*rsmH*	202	52.1327	0.2234929	Ribosomal RNA small subunit methyltransferase H
20	*NA*	202	52.1327	0.2165309	Peptidase S49 family protein

a Shared with previously reported phylogenetic markers for Bacteria [26].

b Shared with previously reported phylogenetic markers for the genus *Staphylococcus* [61].

RFD, Robinson–Fould distance; π, nucleotide diversity; %ShBip, percentage of shared bipartitions with the species tree.

Finally, we tried out the utility of our 13 best markers for searching *Acinetobacter* species in metagenomic data using SRST2 (see Materials and Methods); we tested the markers on the Anacostia river metagenome data set in which the presence of *Acinetobacter* species has been previously reported Anacostia river (Cagle et al. 2019). Notably, we found that all the 13 markers were well covered (above 99.9 of their length, see supplementary table 3, Supplementary Material online) with very low levels of divergence for *A. baumannii* and *Acinetobacterjunii*. Therefore, it seems that our top 13 markers are also well suited for screening *Acinetobacter* species in metagenomes.

## Discussion

The genus *Acinetobacter* is a very versatile group of species, for which it is important to provide a phylogenetic context for any evolutionary analysis. Using a robust phylogenomic approach we inferred the most up-to-date and accurate picture of the phylogenetic relationships for the genus and even singled out suitable phylogenetic markers. The Species Tree allowed us to identify several instances of poor classification, with incorrectly assigned strains, and even one taxon (*A. pittii-*like ANC 4050) that it is very likely to be a new species.

We did not find that any single gene tree matched the Species Tree. This problem in bacteria has been mainly attributed to widespread of Horizontal Gene Transfer (Wolf et al. 2001); however, given that we are dealing with SGF specifically chosen due to vertical inheritance, lack of recombination, and no duplications, it is more likely that topological differences are due to the reduced number of sites analyzed in the gene versus the SGF concatenated alignment (Pamilo and Nei 1988; Haggerty et al. 2009) or incomplete lineage sorting (Castillo-Ramírez and González 2008; Castillo-Ramirez et al. 2010).

Analyzing gene content of different strains and species is very important because gene gain/loss dynamics lies at the center of the theories about the origin and diversification of bacterial species (Cohan 2002); and great importance to gene content and its association with the phenotypic characteristics of a species is expected. Thus, it is highly desirable to have a species delimitation method directly linked to gene composition. We assessed the level of variation in gene composition between *Acinetobacter* spp., in order to establish if these differences can help in species delimitation. We found that using gene content to delineate species it is not reliable for *Acinetobacter* spp. When applying the NJ approach (Euclidean distance + NJ clustering), although we obtained monophyletic groups for many species, when compared with the Species Tree topology, the NJ topology could not retrieve accurately the evolutionary relationships between species. Furthermore, a very conflicting arrangement in the Acb complex species was observed. In a similar way, the PCoA approach did not allow us to distinguish properly between species not only in Acb complex species but also in other species (*A. soli* and *Acinetobacterbaylyi* for example). Our gene content matrix analysis shows that there is considerable variation in gene content within and between the species of this genus, explaining why the NJ approach and the PCoA might be not as useful as the Species Tree to infer the evolutionary history of the genus. Furthermore, the issues with the Acb complex are not unexpected as the species from this group have a rather similar gene content. Acb complex poses a major challenge to this genus, the short branches in the phylogeny and the high level of gene content similarity shown by Acb complex species suggest a very recent diversification of those species. Interestingly, boundaries for gene exchange appear to be very flexible within the Acb complex clade and probably homologous recombination events can be contributing to homogenize gene composition as well. The grouping established by the presence–absence of genes in the genomes under consideration can reflect the existence of genetic barriers to recombination and the rate of Horizontal Gene Transfer between species (Shapiro and Polz 2014); in this sense, gene content comparison is a very valuable tool for analyzing evolutionary processes underlying species diversification but it is not reliable for species assignation when those rates and bounds of genetic variation can be very different between species in the same genus.

Species assignation issues in *Acinetobacter* are evident, especially for the Acb complex clade. The rapid and precise identification of Acb species is very relevant from a clinical point of view; thus, it is very important to have rapid and reliable methods for the allocation of bacterial species. Species assignation methods based on whole genome surveys, such as DDH or ANI, are ideal but DDH is methodologically laborious and ANI requires WGS, which can be costly and nonpractical when dealing with large samples. The search for appropriate phylogenetic markers for species assignation in Bacteria has been going for decades. Among the most frequently used markers (not only for *Acinetobacter* but for many other bacterial species) are *rpoB, gyrB*, and *recA*. However, here we show that at least for this genus these are not the best candidates. The multi locus sequence analysis (MLSA) approach has been proposed to replace the DDH technique and even a similarity percentage based on the concatenated alignment of MLSA genes has been proposed (Rong et al. 2009). But this scheme has also been criticized mainly for two reasons: The arbitrariness in the choice of the genes and the possibility of great variation between genera (Chan et al. 2012). Our work, this study and a recent article about the best markers for the genus *Staphylococcus* (Graña-Miraglia et al. 2018), strongly supports those two issues. On the other hand, other phylogenetic markers proposed previously for Bacteria by Zeigler (2003) were found to be amongst the group of the 255 SGFs. These are, according to the functional annotation, 13 genes (*recN, ruvB, dnaJ, ffh, atpA, tig, rho, recA, rpoA, lepA, ftsZ, dxs*, and *pgk)* with very variable functions and widely spread in our ranking. However, only one of these genes is in the top 20 ranked genes (*dxs*, in 11th position). We also found that 10 of our 255 SGF are shared with a group of phylogenetic markers proposed for the 3 domains of life (Ciccarelli et al. 2006), most of them corresponding to ribosomal proteins. Moreover, when we compared our 255 SGFs with the recently described 177 best makers for the genus *Staphylococcus* by our group (Graña-Miraglia et al. 2018), we obtained just 11 genes in common (see supplementary table 2, Supplementary Material online). All these facts support the notion that there are very few potential universals markers. However, this study and our recent report study on the genus *Staphylococcus* clearly suggest that one can identify phylogenetic markers for optimal bacterial species classification in specific genera. Notably, these markers can be used for pathogen detection from environmental samples.

We have chosen our phylogenetic markers using explicit evolutionary criteria and just to be used as specific markers for the genus *Acinetobacter*, these genes probed to distinguish between different species with high fidelity. Undoubtedly, WGS is the best strategy to infer the evolutionary relationships; however, if WGS is not affordable, we propose to use various loci to assign species within the genus *Acinetobacter*, according to our study this approach offer high resolution for species assignation. In this regard, we proved that the concatenation of the top 13 markers is enough to increase topological similarity almost to the level of WGS. Clearly, this increase in phylogenetic power is not only because of the larger number of sites being analyzed but also due to the genes chosen, as we tried sets of randomly chosen genes and almost none of them showed the high similarity to the Species Tree as the top markers (fig. 4). Theoretically, these top 13 SGFs are the ideal candidates for developing an MLSA for the genus. However, practical issues (primer design, for instance) should be taken into account; nonetheless, even if some of these were to show experimental drawbacks, those can be replaced by the following genes in the list. Summarizing, we highlight decent phylogenetic markers for reconstructing the phylogeny of the genus and these markers appear to be good phylogenetic markers even for screening species of this genus in metagenomes.

## Conclusion

On the whole, this study gives the latest view of the phylogenetic relationships for the *Acinetobacter* spp. (showing several cases of poor classification) and unveils a list of well-suited genes for species assignation in the genus *Acinetobacter*. We anticipate that this sort of bespoke phylogenomic strategies will become the norm for many other bacterial genera in the next decades.

## Availability of Data and Materials

The authors declare that the data supporting the findings of this study are available within the article and its supplementary information files.

## Supplementary Material

Supplementary data are available at *Genome Biology and Evolution* online.

## Supplementary Material

evz178_Supplementary_Data
