# Supplementary figures and images for: Phylogenomics Reveals Clear Cases of Misclassification and Genus-Wide Phylogenetic Markers for Acinetobacter
Source: Genome Biol Evol. 2019 Aug 12;11(9):2531–41. doi: 10.1093/gbe/evz178 (PMC6740150; doi:10.1093/gbe/evz178)

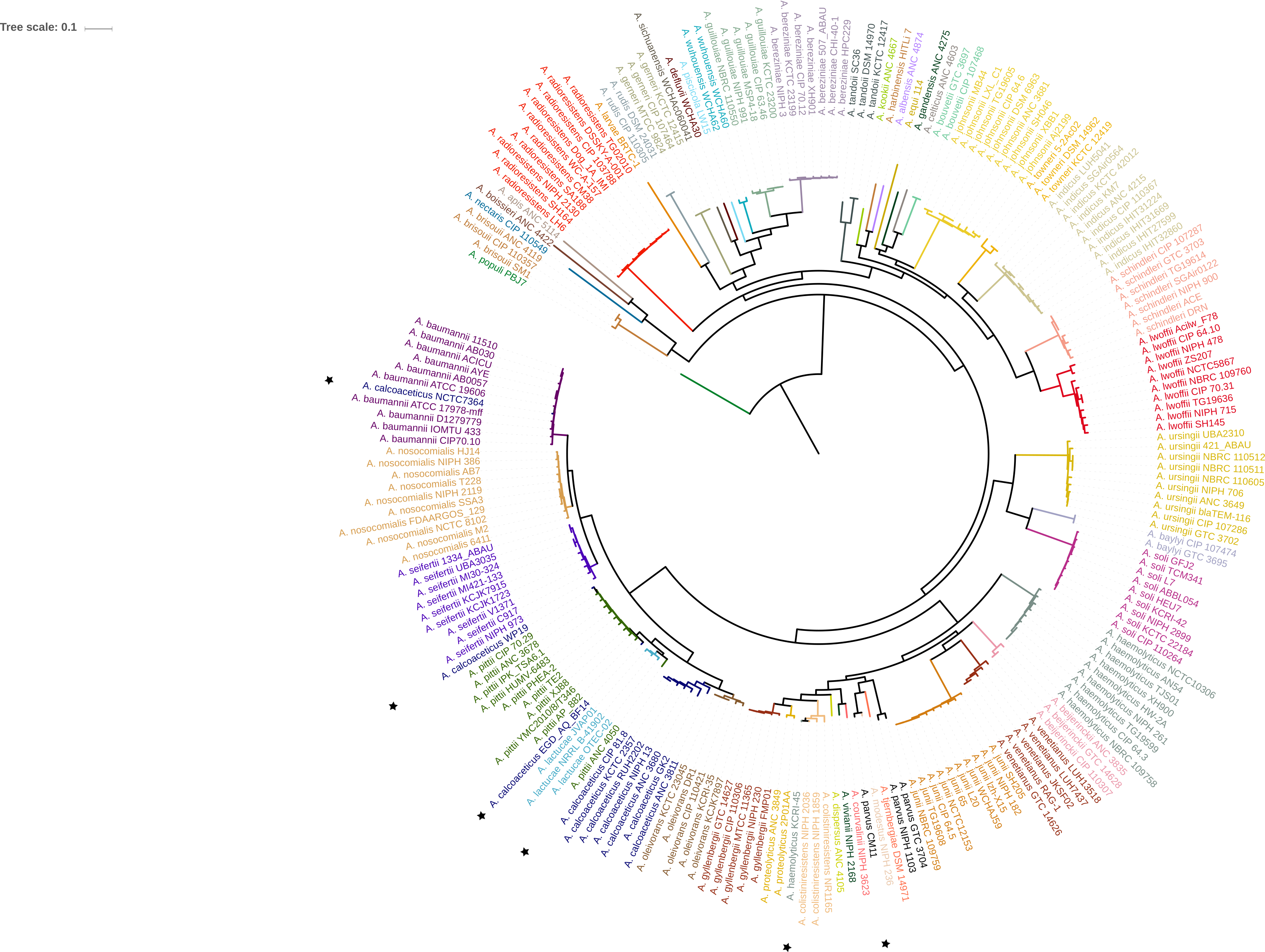

Supplement: evz178_Supplementary_Data [file evz178_supplementary_data.zip › Supplementary_Figure1.tiff]

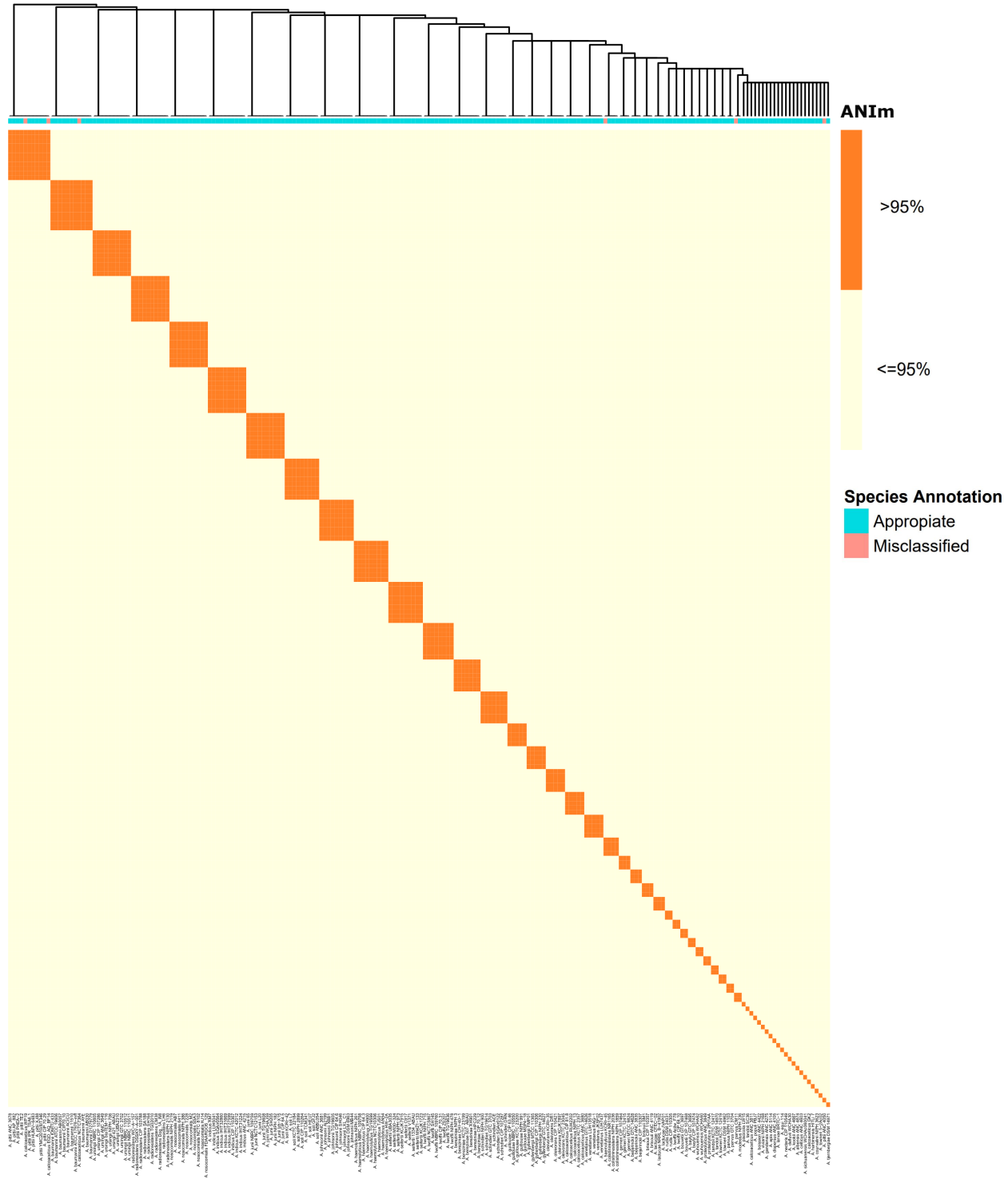

Supplement: evz178_Supplementary_Data [file evz178_supplementary_data.zip › Supplementary_Figure2.pdf]

## Acb Complex

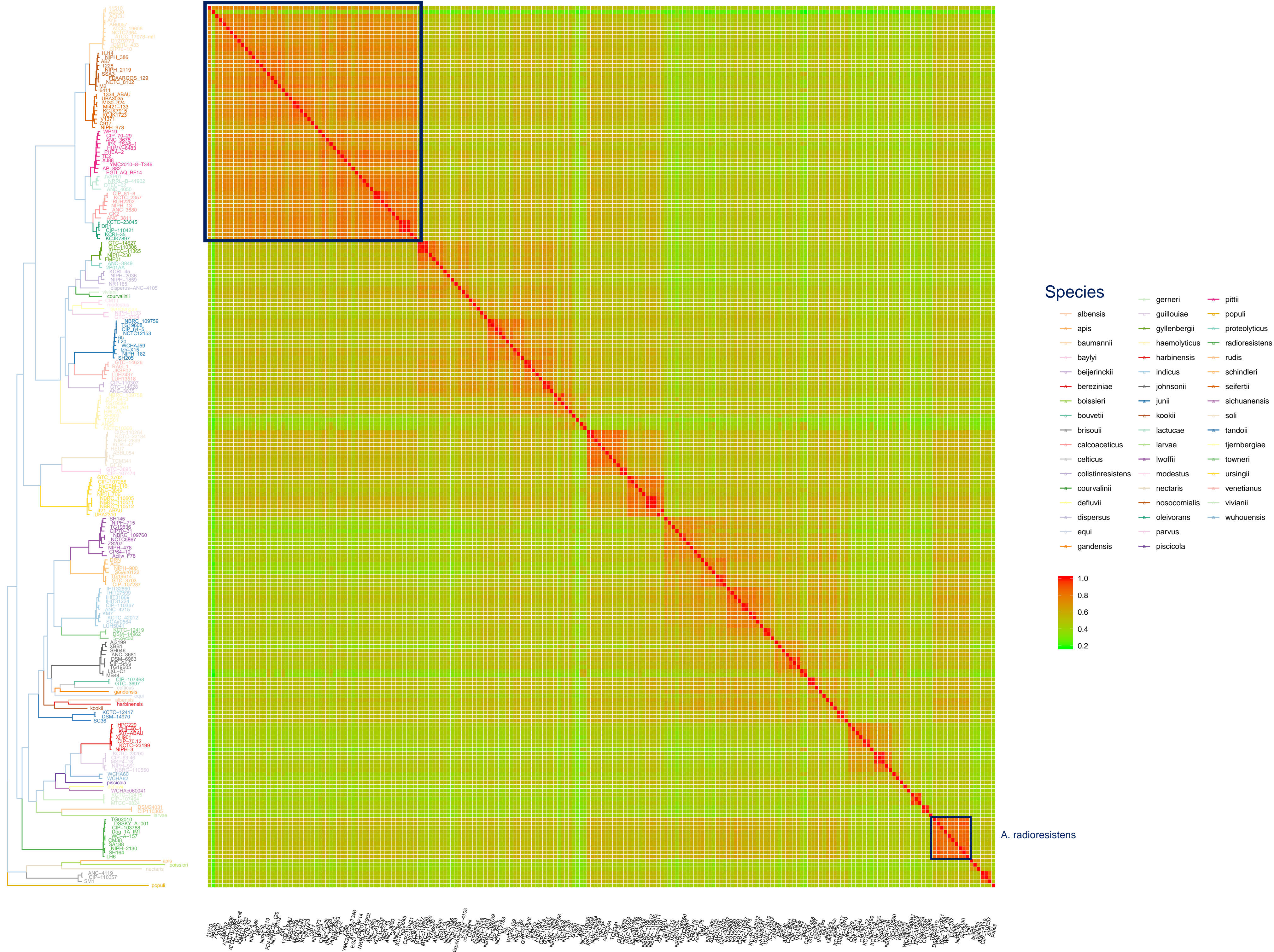

Supplement: evz178_Supplementary_Data [file evz178_supplementary_data.zip › Supplementary_Figure3.pdf]
