# Supplementary material for: Phylogenomics Reveals Clear Cases of Misclassification and Genus-Wide Phylogenetic Markers for Acinetobacter
Source: Genome Biol Evol. 2019 Aug 12;11(9):2531–41. doi: 10.1093/gbe/evz178 (PMC6740150; doi:10.1093/gbe/evz178)
Supplement: evz178_Supplementary_Data [file evz178_supplementary_data.zip › Legends suppl materials.doc]

**Supplementary material**

**Supplementary Figure 1**. Species tree for the genus *Acinetobacter*. Same tree as in Figure 1A but in a circular display and showing a higher resolution. Black asterisks point out misclassified strains. Tree scale shows substitutions per site.

**Supplementary Figure 2**. Heat map of the average nucleotide identity of 214 *Acinetobacter* genomes. The orange cells represent values above 95% of similarity, indicating that two organisms belong to the same species; otherwise, the cells are colored in yellow. The top row distinguishes between properly classified (blue cells) or misclassified strains (reddish cells). The dendogram across the top depicts the strain clustering.

**Supplementary Figure 3**. Heat map of the gene content correlation matrix for the genus *Acinetobacter*. The tree on the left-hand side shows the clustering as in Figure 1A. Color-coding is explained on the right-hand side and gives the different species. The purple rectangle depicts the Acb complex. The labels on the tree and at the bottom are the strain identifiers as per Supplementary Table 1.

**Supplementary Table 1**. Database of the 214 *Acinetobacter* publicly available genomes included in our study. The first columns contain the NCBI genome statistics for each organism. Rows in bold correspond to type strains as reported in LPSN (http://www.bacterio.net/index.html). The last two columns are the CHECKM values for completeness and contamination (above 95% and less than 5% respectively.

**Supplementary Table 2**. List of the orthologous genes found in this study. The 255 non-recombinant SGF were annotated with Uniprot. The first column has the short name of the gene. The second and the third column have the Robinson-Fould Distance (RFD) and percentage of Shared Bipartitions (ShBip) for each SGF with the species tree, respectively. The fourth and the fifth column have information of the Nucleotide Diversity (ND) and Uniprot gene annotation of each SGF. The asterisks in the first column distinguish the genes with ambiguous annotation. The superscripts indicate the reference where the gene was described before as a phylogenetic marker: 1 indicates that the gene was previously used as a phylogenetic marker for Bacteria (Zeigler 2003); 2 if it was described as a universal marker (Ciccarelli et al. 2006); and 3 evidence the genes recently reported as phylogenetic markers for the genus Staphylococcus (Graña-Miraglia et al., 2018).
